# Prevalence of Headache Days and Disability 3 Years After Participation in the Childhood and Adolescent Migraine Prevention Medication Trial

**DOI:** 10.1001/jamanetworkopen.2021.14712

**Published:** 2021-07-12

**Authors:** Scott W. Powers, Christopher S. Coffey, Leigh A. Chamberlin, Dixie J. Ecklund, Elizabeth A. Klingner, Jon W. Yankey, James L. Peugh, Leslie L. Korbee, Kerry Simmons, Stephanie M. Sullivan, Marielle A. Kabbouche, Joanne Kacperski, Linda L. Porter, Brooke L. Reidy, Andrew D. Hershey

**Affiliations:** 1 Division of Behavioral Medicine and Clinical Psychology, Cincinnati Children’s Hospital Medical Center, Cincinnati, Ohio; 2 Cincinnati Children’s Headache Center, Cincinnati, Ohio; 3 Department of Pediatrics, University of Cincinnati College of Medicine, Cincinnati, Ohio; 4 Department of Biostatistics, Clinical Trials Statistical and Data Management Center, University of Iowa College of Public Health, Iowa City; 5 Academic Regulatory & Monitoring Services, Cincinnati, Ohio; 6 Office for Clinical and Translational Research, Cincinnati Children’s Hospital Medical Center, Cincinnati, Ohio; 7 Division of Neurology, Cincinnati Children’s Hospital Medical Center, Cincinnati, Ohio; 8 National Institute of Neurological Disorders and Stroke, Bethesda, Maryland

## Abstract

**Importance:**

Migraine is a common neurological disease that often begins in childhood and continues into adulthood; approximately 6 million children and adolescents in the United States cope with migraine, and many frequently experience significant disability and multiple headache days per week. Although pharmacological preventive treatments have been shown to offer some benefit to youth with migraine, additional research is needed to understand whether and how these benefits are sustained.

**Objective:**

To survey clinical status of youth with migraine who participated in the 24-week Childhood and Adolescent Migraine Prevention (CHAMP) trial over a 3-year follow-up period.

**Design, Setting, and Participants:**

This survey study used internet-based surveys collected from youth ages 8 to 17 years at 3, 6, 12, 18, 24, and 36 months after completion of the CHAMP trial, which randomized participants to amitriptyline, topiramate, or placebo. At the end of the trial, the study drug was stopped, and participants received clinical care of their choice thereafter. The CHAMP trial was conducted between May 2012 and November 2015, and survey follow-up was conducted June 2013 to June 2018. Participants in this survey study were representative of those randomized in the trial. Data were analyzed from March 2020 to April 2021.

**Exposures:**

Survey completion.

**Main Outcomes and Measures:**

Headache days, disability (assessed using the Pediatric Migraine Disability Scale [PedMIDAS]), and self-report of ongoing use of prescription preventive medication.

**Results:**

A total of 205 youth (mean [SD] age, 14.2 [2.3] years; 139 [68%] girls; mean [SD] history of migraine, 5.7 [3.1] years) participated in the survey. Retention of participants was 189 participants (92%) at month 6, 182 participants (88%) at month 12, 163 participants (80%) at month 18, 165 participants (80%) at month 24, and 155 participants (76%) at month 36. Over the course of the 3-year follow-up, participants consistently maintained meaningful reductions in headache days (mean [SD] headache days per 28 days: CHAMP baseline, 11.1 [6.0] days; CHAMP completion, 5.0 [5.7] days; 3-year follow-up, 6.1 [6.1] days) and disability (mean [SD] score: CHAMP baseline, 40.9 [26.4]; CHAMP completion, 17.9 [22.1]; 3-year follow-up, 12.3 [20.0]). At 3 years after completion of the CHAMP trial, headache days were approximately 1.5 per week (changed from about 3 per week at trial baseline) and disability had improved from the moderate range to the low mild range on the PedMIDAS. Longitudinal analyses showed that amitriptyline and topiramate did not explain intercept random effects for either mean rate of headache days per week (amitriptyline: estimate [SE], 0.07 [0.05]; *P* = .16; topiramate: estimate [SE], 0.04 [0.05]; *P* = .50) or headache disability PedMIDAS total score (amitriptyline: estimate [SE], 0.25 [0.38]; *P* = .52; topiramate: estimate [SE], −0.09 [0.39]; *P* = .82) changes over time. Of 153 participants who reported on prescription drug use at 3 years, only 1 participant (1%) reported using prevention medication, and most participants reported no medication use at most time points.

**Conclusions and Relevance:**

These findings suggest that children and adolescents with longer than 5 years history of migraine who participated in the CHAMP trial may sustain positive clinical outcomes over time, even after discontinuing preventive pill-based treatment. This survey study could inform use and discontinuation timing of pharmacological preventive therapies for migraine in youth ages 8 to 17 years. Research is needed to examine mechanisms of treatment improvement and maintenance for preventive therapies, as well as placebo, in the pediatric population.

## Key Points

**Question:**

How do children and adolescents with migraine function after participating in a multicenter migraine prevention medication trial?

**Findings:**

This survey study that followed up 205 participants from the Childhood and Adolescent Migraine Prevention study at 3, 6, 12, 18, 24, and 36 months found that regardless of treatment group (amitriptyline, topiramate, placebo), youth consistently maintained meaningful reductions of headache days and migraine-related disability for 3 years after completion of the trial. Less than 10% of the participants reported ongoing use of prevention medication prescribed as part of typical clinical care, with most participants reporting no medication use at most time points.

**Meaning:**

These findings suggest that children and adolescents may get better and sustain positive outcomes over time without ongoing pharmacotherapy.

## Teaser

This survey study assesses migraine prevalence and disability of participants who completed the Childhood and Adolescent Migraine Prevention trial during 3 years of follow-up.

## Introduction

Migraine is a common neurological disease that often begins in childhood and persists into adulthood.^1,2,3,4^ It is the second most disabling disease in the world, yet it is underrecognized, undertreated, and underappreciated.^5,6,7,8,9^ For people ages 15 to 49 years, migraine is the top cause of years lived with disability.^10,11^ Because there are few rigorous pediatric trials,^12,13^ clinical care for youth with migraine is primarily based on expert opinion and extrapolation of adult medication trial data.^11,14,15^ The dearth of evidence for pediatric patients motivated the Childhood and Adolescent Migraine Prevention (CHAMP) trial (NCT01581281),^16^ a multicenter study funded by the National Institutes of Health. The CHAMP trial compared the 2 most commonly used medications in current practice, amitriptyline^17^ and topiramate,^12,18,19^ with a placebo and with each other. The trial included youth ages 8 to 17 years with migraine (migraine without aura, migraine with aura, and chronic migraine) who reported between 4 and 28 headache days during the 28-day baseline and had at least mild levels of disability. The trial protocol, baseline data, and primary results have been published previously.^14,16,20,21^

The CHAMP trial was stopped early owing to futility. Results showed no differences between the amitriptyline, topiramate, or placebo groups in terms of the proportion of participants with a 50% or greater reduction in headache days, reduction in participants’ absolute number of headache days, or changes in participants’ levels of migraine-related disability when data from a 28-day baseline period were compared with the final 28 days of a 24-week treatment period.^16^ Most participants in each group exhibited clinically meaningful and significant reductions in headache days and disability (eg, 6 to 7 fewer headache days per month from a baseline of about 12 headache days per 28 days).^16^ A critically important question for understanding longer-term prognosis and guiding clinical care^15,22^ was what about after 24 weeks?

This survey study is a follow-up of the CHAMP trial examining the status of participants in terms of headache days, disability, and current use of any prescription prevention medications at 3, 6, 12, 18, 24, and 36 months after the end of the trial. We investigated 4 main questions. First, were survey participants representative of the enrolled participants in the CHAMP trial, and how well were we able to retain participants for 3 years? Second, within each study group, did youth maintain improvements in headache days and disability from baseline over a 3-year period after the end of the CHAMP trial? Third, did any differences in outcomes arise over 3 years of follow-up among the amitriptyline, topiramate, and placebo groups? And fourth, how many survey participants were using prescription prevention medication during the 3-year follow-up period? Guided by findings from a 1, 2, and 5-year follow-up study of standard clinical care at the Cincinnati Children’s Headache Center,^23^ we hypothesized that treatment gains from baseline would be maintained over time in these follow-up survey participants.

## Methods

This survey study was approved by the Cincinnati Children’s institutional review board. All participants provided electronic informed consent and/or assent. Our study approach was informed by the American Association for Public Opinion Research (AAPOR) reporting guideline. Specifically, we had specific goals, selected a sample that represented the population of interest, used a design that balanced costs with errors, trained interviewers carefully, checked quality regularly, maximized response rates within ethical standards, and used appropriate statistical techniques.

### Participants

Between May 2012 and November 2015, a total of 264 youth participated in the CHAMP trial and had a final treatment visit at which end point data were obtained. A complete list of CHAMP inclusion and exclusion criteria can be found elsewhere.^16^ Participants who initiated survey questionnaires at 3 months were considered the group that would be asked to continue to provide data at future time points.

Once the final trial visit occurred, participants could continue clinical care at the study site, change to another health care practitioner, stop seeing a clinician and pursue self-care, or stop care and then return. No study drug was provided after the end of the CHAMP trial. Any subsequent prescriptions of preventive medications, including the agent(s), dosing, and duration of treatment, occurred as part of clinical care of the participant’s choosing.

### Recruitment

Within 3 months of completion of the CHAMP trial, participants were recruited to enroll in the follow-up survey. Families at each trial site (except 1 site, which included 4 participating families) had previously provided parental permission allowing recontact for future studies. During the final in-person study visit for the CHAMP trial, the study coordinator at each participating site provided an overview of the follow-up survey and confirmed the family’s willingness for recontact. Families who agreed were contacted by telephone and provided with clear instructions about accessing the online survey, along with a password. After their questions were answered, they accessed a secure website and completed electronic informed consent and/or assent.

### Study Design and Procedures

#### Data Collection

From June 2013 to June 2018, participants used a secure server to answer internet-based questionnaires at preset time points. The questionnaires required approximately 20 minutes to complete. Participants completed them at their own convenience within these time intervals: month 3, within 2 to 5 months after trial completion; month 6, within 5 to 8 months after trial completion; month 12, within 12 to 14 months after trial completion; month 18, within 18 to 20 months after trial completion; month 24, within 24 to 28 months after trial completion; and month 36, within 36 to 40 months after trial completion. Youth were asked to complete the tasks either independently or with the assistance of a parent or caregiver. Participants who completed the 3-month questionnaires and did not complete future questionnaires within the specified time interval were recontacted and encouraged to complete the questionnaires during the next time point. In the CHAMP trial, participants and investigators remained blinded to treatment assignment after completion of the 24-week intervention period. They were notified of the trial outcomes and group assignment once the study database was locked for analyses. This did not occur for any trial participant or site investigator until after the completion of the 12-month survey time point.

#### Retention

Strategies used to maximize retention of participants included the social media, telephone contact, newsletters, and personalized mailings. Participants were reimbursed for their time and effort using a debit card that was mailed to their household. Reimbursements of $15 to $20 were added to this card immediately on completion of the questionnaires for a given time point.

### Measures

#### Headache Frequency

Self-reported headache frequency was assessed using responses to the question, “How many headaches have you had in the last 4 weeks (28 days)?” Participants recorded their recollection as number of days. Of note, comparisons in a 2013 clinical trial^24^ demonstrated that this approach was highly correlated with diary records in a sample of 104 youth ages 10 to 17 years with migraine who had been keeping a daily diary over several months, similar to the experience of CHAMP participants (*r* = 0.90).

#### Migraine Disability

Headache-related disability was assessed through the Pediatric Migraine Disability Assessment Scale (PedMIDAS), a standardized, reliable, and validated disability questionnaire specific to youth with migraine.^25^ The PedMIDAS was designed to be administered as a semistructured interview, measuring the impact of headaches on day-to-day school, home, play, and social activities over the last 3 months. To obtain these data via the internet, the PedMIDAS relied on self-reported data. Of note, all participants had familiarity with PedMIDAS, and had completed it in an interview 3 times during the CHAMP trial. PedMIDAS scores can range from 0 to 240. The clinical grading scale for PedMIDAS is I, little to no disability (score, 0-10); II, mild level (score, of 11-30); III, moderate level  (score, 31-50); and IV, severe level (score, >50).^26^

#### Prescription Medications Used for Headache Prevention

Participants were provided a broad list of prescription prevention therapies for headache. They were asked to select any and all prescription medications used to prevent headaches over the previous 3 months.

### Statistical Analysis

Participant demographic characteristics were compared across 3 groups: those enrolled in the CHAMP follow-up survey, those who were eligible for the survey but did not enroll, and those who were ineligible (ie, did not attend a final trial study visit and provide end point data). Data summaries of primary outcome data were generated across the 3 treatment groups assigned during the original CHAMP trial. Outcome data included headache days, headache disability (as measured by PedMIDAS), and the proportion of CHAMP follow-up survey participants reporting use of prescription prevention medications. To present this information, continuous variables were summarized by means and SDs, while categorical variables were summarized by frequencies and percentages. Analyses for headache days and headache disability were conducted in 2 ways.

In our longitudinal approach, longitudinal growth curve analyses were conducted, keeping 3 model specifications in mind. First, participants were assessed on an unequal interval schedule (ie, 3-, 6-, 12-, 18-, 24-, and 36-month time points), but the metric used to measure response variable changes in analysis must be consistent for interpretational ease. To accomplish this, linear slope loadings for the growth curve models were given values of 0, 1, 3, 5, 7, and 11 (loadings for higher-order trend components were accomplished by applying the appropriate powered vectors to the linear slope loadings) so that interpretations of linear slope and other higher order trend components could be interpreted as the expected response variable change per 3-month increment of time. Second, longitudinal response variable data were analyzed using model-building techniques^27^ to ensure proper specification. Specifically, trajectory (eg, linear, quadratic, cubic) fixed effects (or means) were added to the longitudinal model if statistically significant. However, fixed effects that were not statistically significant were retained in the longitudinal model if their respective corresponding random effect (or variance estimate) was statistically significant. This process continued until statistically significant trend component fixed or random effects could no longer be added to the longitudinal model. However, the response variable headache day was modeled longitudinally as count data. Preliminary sensitivity analyses conducted prior to model building in the second step showed a negative binomial model was needed owing to statistically significant dispersion parameter estimates for 5 of the 6 observed longitudinal assessments. In the final step, and after the best-fitting model was specified in the second step, the amitriptyline and topiramate independent variable groups (placebo was the reference group) were dummy-coded and included in both longitudinal growth models as time-invariant independent variables.

In our per follow-up time point approach, results from the headache days and headache disability comparisons are presented as means with SE estimates across the 6 follow-up assessment time points. Analyses for headache days and headache disability were conducted at the 3-month (to assess changes immediately after completion of the CHAMP trial) and 6-, 12-, 18-, 24-, and 36-month (to assess longer term changes after trial participation) follow-up time points. The family-wise type-1 error rate across the 18 pairwise comparisons (3 independent variable groups × 6 assessment time points) was controlled for using the false discovery rate (FDR),^28^ which was calculated separately for headache days pairwise comparisons and headache disability pairwise comparisons.

Comparisons of percentages of participants using medications at the 3-, 6-, 12-, 18-, 24-, and 36-month follow-up visits were performed using Pearson χ^2^ tests. Specifically, all 3 possible between independent variable group comparisons (ie, placebo vs amitriptyline, placebo vs topiramate, and amitriptyline vs topiramate) were conducted at all follow-up time points. The family-wise type-1 error rate across the 18 pairwise comparisons (3 independent variable groups × 6 assessment time points) was controlled for using the FDR.^28^

All analyses were performed using SPSS version 24 (IBM) and Mplus (version 8.4) statistical software. *P* values were 2-tailed and *P* < .05 constituted statistically significant findings for all analyses. Data were analyzed from March 2020 to April 2021

## Results

### Participant Characteristics

Of 264 eligible participants, 251 were contacted, and 215 families (86%) representing 25 CHAMP trial sites across the United States agreed to participate. Of these, data were not obtained from 10 families (5%) who were no longer interested in participating after having consented to participate. Thus, 205 youth (mean [SD] age, 14.2 [2.3] years; 139 [68%] girls) completed the 3-month follow-up survey questionnaires. Participants reported a mean (SD) history of headaches of 5.7 (3.1) years. All participants had been diagnosed with migraine using standardized international classification criteria^29^ by a site investigator physician. They had a mean (SD) of 11.1 (6.0) headache days over the course of the 28-day baseline assessment, with 49 participants (24%) having chronic migraine (Table 1). Overall, participants completing the CHAMP follow-up survey showed no demographic or trial outcome differences compared with CHAMP trial participants who were not eligible or chose not to participate in the follow-up survey; this indicated that the characteristics of the participants in the CHAMP follow-up survey were representative of all CHAMP trial participants. At each time interval, the sample remained representative of participants who initially consented for the trial. The retention rate of participants, as well as data completion, remained high at each time interval in the follow-up survey: 189 participants (92%) at month 6, 182 participants (88%) at month 12, 163 participants (80%) at month 18, 165 participants (80%) at month 24, and 155 participants (76%) at month 36.

**Table 1.** Demographic Characteristics of Enrolled vs Nonenrolled CHAMP Follow-up Survey Participants

Variable	No. (%)		
	CHAMP follow-up survey		Not eligible for CHAMP follow-up survey (n = 97)^a^
	Enrolled (n = 205)	Eligible but not enrolled (n = 59)	
Age, mean (SD), y^b^	14.2 (2.3)	14.3 (2.6)	14.2 (2.4)
Sex			
Girls	139 (68)	44 (75)	64 (66)
Boys	66 (32)	15 (25)	33 (34)
Race/ethnicity^c^			
White	143 (70)	41 (69)	69 (71)
Not White	60 (29)	16 (27)	25 (26)
Not known or not reported	2 (1)	2 (3)	3 (3)
Not Hispanic/Latino^c^	182 (89)	50 (85)	84 (87)
Maternal education			
<High school	5 (2)	2 (3)	5 (5)
High school diploma	28 (14)	8 (14)	15 (15)
Some college or technical school	69 (34)	20 (34)	28 (29)
College degree	72 (35)	26 (44)	37 (38)
Graduate degree	22 (11)	2 (3)	8 (8)
Unknown	9 (4)	1 (2)	4 (4)
Paternal education			
<High school	7 (3)	3 (5)	6 (6)
High school diploma	41 (20)	12 (20)	32 (33)
Some college or technical school	70 (34)	16 (27)	13 (13)
College degree	52 (25)	21 (36)	24 (25)
Graduate degree	22 (11)	1 (2)	12 (12)
Unknown	13 (6)	6 (10)	10 (10)
Annual family income, $			
<20 000	17 (8)	5 (8)	13 (13)
20 000-34 999	20 (10)	7 (12)	16 (16)
35 000-49 999	16 (8)	8 (14)	7 (7)
50 000-74 999	38 (19)	11 (19)	15 (15)
75 000-99 999	33 (16)	4 (7)	15 (15)
100 000-149 999	37 (18)	7 (12)	15 (15)
≥150 000	13 (6)	1 (2)	8 (8)
Not reported or unknown	31 (15)	16 (27)	8 (8)
Headache d/mo, No.			
Baseline	11.1 (6.0)	11.6 (5.9)	12.0 (6.6)
Wk 24	5.0 (5.7)	3.7 (3.4)	
PedMIDAS disability score			
Baseline	40.9 (26.4)	42.1 (26.7)	43.8 (27.9)
Wk 24	17.9 (22.1)	14.8 (17.1)	
History of headache, mean (SD), y	5.7 (3.1)	5.8 (3.3)	5.1 (3.1)

Abbreviations: CHAMP, Childhood and Adolescent Migraine Prevention; PedMIDAS, Pediatric Migraine Disability Scale.

^a^ To be eligible, a CHAMP participant needed to complete the final study visit and provide end point data while continuing study drug use so that end-of-trial status could be compared with follow-up reports. In addition, since the CHAMP trial was stopped early owing to futility, some participants could not continue to the final study visit. These participants were also not eligible for the CHAMP follow-up survey.

^b^ Computed based on the date of enrollment in the CHAMP study.

^c^ Race and ethnicity were reported by patient or surrogate. Participants in the not White race category included Black, Asian, American Indian or Alaskan Native, Native Hawaiian or Pacific Islander.

### Headache Days per 28-Day Period

At the end of the 3-year follow-up, participants experienced a mean (SD) of 6.1 (6.1) headache days per month, compared with 11.1 (6.0) headache days per month at baseline and 5.0 (5.7) headache days per month at the end of the CHAMP trial (Table 2). The Figure presents findings from the longitudinal analyses. We found that amitriptyline and topiramate did not significantly explain intercept (amitriptyline: estimate [SE], −0.05 [0.16]; *P* = .73; topiramate: estimate [SE], 0.11 [0.16]; *P* = .51), linear slope (amitriptyline: estimate [SE], 0.07 [0.05]; *P* = .16; topiramate: estimate [SE], 0.04 [0.05]; *P* = .50), or quadratic change (amitriptyline: estimate [SE], −0.004 [0.004]; *P* = .30; topiramate: estimate [SE], −0.004 [0.005]; *P* = .35) random effects for changes in mean headache day rate over time.

**Table 2.** Descriptive Statistics and Statistical Analyses for Headache Days

Time point	Headache d/mo, mean (SD)				Placebo vs amitriptyline			Placebo vs topiramate			Amitriptyline vs topiramate		
	Overall	Amitriptyline	Topiramate	Placebo	Estimate, mean (SE)	*P* value	FDR	Estimate, mean (SE)	*P* value	FDR	Estimate, mean (SE)	*P* value	FDR
CHAMP study													
Baseline	11.1 (6.0)	11.2 (6.2)	11.2 (5.9)	10.8 (5.8)	NA	NA	NA	NA	NA	NA	NA	NA	NA
End	5.0 (5.7)	4.9 (5.0)	4.7 (5.6)	5.9 (7.1)	NA	NA	NA	NA	NA	NA	NA	NA	NA
3-mo	6.4 (5.9)	6.2 (5.7)	6.7 (6.7)	6.1 (5.1)	−0.092 (0.987)	.93	0.050^a^	−0.533 (1.064)	.62	0.031^a^	−0.442 (0.992)	.66	0.036^a^
6-mo	6.8 (6.8)	6.2 (6.5)	7.6 (7.1)	6.5 (7.0)	0.304 (1.287)	.81	0.044^a^	−1.062 (1.333)	.43	0.022^a^	−1.367 (1.132)	.23	0.014^a^
12-mo	7.1 (6.9)	6.9 (6.6)	8.3 (7.7)	5.3 (6.3)	−1.620 (1.236)	.19	0.011^a^	−2.954 (1.329)	.03	0.006^a^	−1.334 (1.206)	.27	0.017^a^
18-mo	6.5 (6.2)	6.7 (6.5)	6.4 (6.1)	6.1 (5.9)	−0.620 (1.262)	.62	0.033^a^	−0.239 (1.235)	.85	0.047^a^	0.381 (1.115)	.73	0.039^a^
24-mo	5.9 (5.9)	6.6 (6.6)	6.0 (5.8)	4.2 (4.4)	−2.392 (1.057)	.02	0.003^a^	−1.744 (1.036)	.09	0.008^a^	0.648 (1.057)	.54	0.025^a^
36-mo	6.1 (6.1)	6.5 (6.6)	5.9 (7.0)	5.4 (5.9)	−1.114 (1.251)	.37	0.019^a^	−0.438 (1.351)	.75	0.042^a^	0.677 (1.251)	.59	0.028^a^

Abbreviations: CHAMP, Childhood and Adolescent Migraine Prevention; FDR, false discovery rate; NA, not applicable.

^a^ *P* value greater than FDR.

**Figure.**
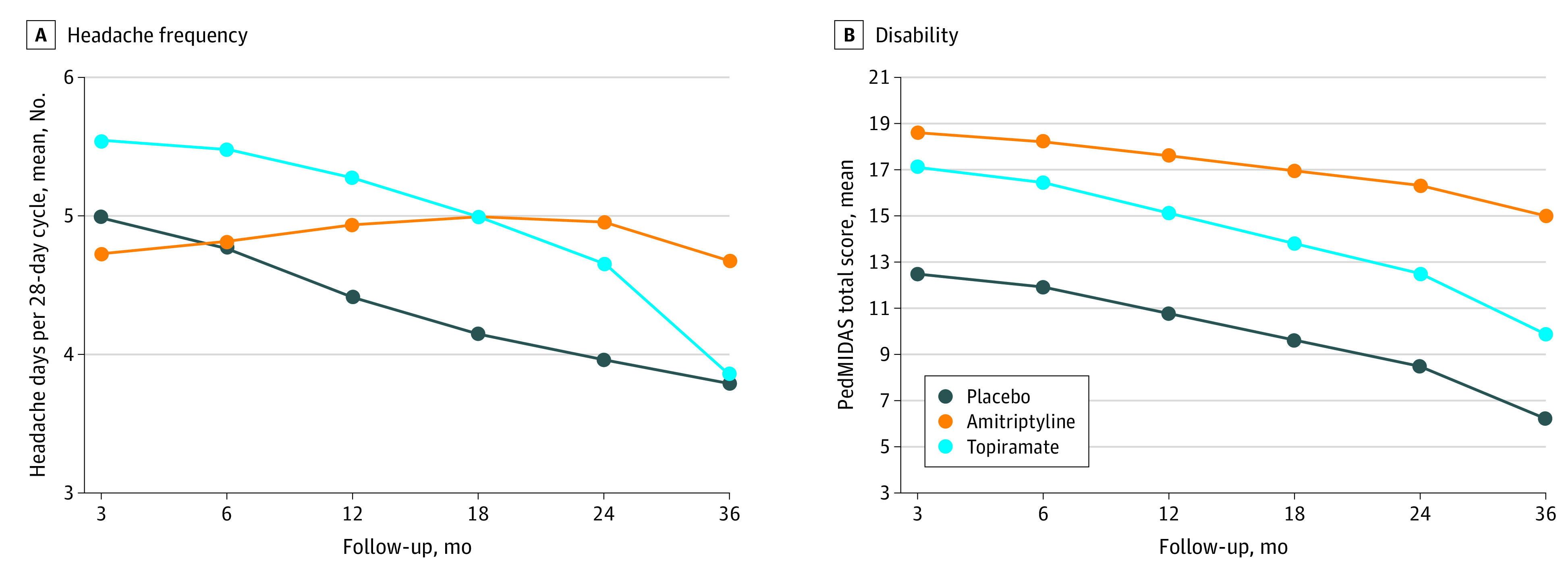
Results From Longitudinal Growth Curve Analyses at 3, 6, 12, 18, 24, and 36 Months for Headache Days and Disability Headache disability was measured by Pediatric Migraine Disability Scale (PedMIDAS).

Table 2 presents complete headache day data by study group and for the overall follow-up survey sample. After FDR was applied, no statistically significant results were observed.

### Headache Disability

At the end of the 3-year follow-up, the mean (SD) overall PedMIDAS disability score was 12.3 (20.0), compared with 40.9 (26.4) at baseline and 17.9 (22.1) at the end of the CHAMP trial (Table 3). Longitudinal analyses showed amitriptyline and topiramate did not significantly explain intercept (amitriptyline: estimate [SE], 6.11 [3.93]; *P* = .12; topiramate: estimate [SE], 4.66 [3.95]; *P* = .24) or linear slope (amitriptyline: estimate [SE], 0.25 [0.38]; *P* = .52; topiramate: estimate [SE], −0.09 [0.39]; *P* = .82) random effects for PedMIDAS total score changes over time (Figure).

**Table 3.** Descriptive Statistics and Statistical Analyses for Migraine Disability by Time Point and Group

Time point, mo	PedMIDAS score, mean (SD)				Placebo vs amitriptyline			Placebo vs topiramate			Amitriptyline vs topiramate		
	Overall	Amitriptyline	Topiramate	Placebo	Estimate, mean (SE)	*P* value	FDR	Estimate, mean (SE)	*P* value	FDR	Estimate, mean (SE)	*P* value	FDR
CHAMP study													
Baseline	40.9 (2.64)	40.9 (28.6)	39.0 (24.9)	44.0 (25.1)	NA	NA	NA	NA	NA	NA	NA	NA	NA
End	17.9 (22.1)	19.4 (25.6)	15.0 (18.9)	20.0 (20.5)	NA	NA	NA	NA	NA	NA	NA	NA	NA
3	17.0 (21.7)	19.7 (25.1)	17.0 (21.4)	12.4 (14.2)	−7.310 (3.546)	.04	0.011^a^	−4.635 (3.188)	.15	0.022^a^	2.674 (3.708)	.47	0.042^a^
6	16.2 (25.0)	16.2 (26.7)	18.4 (27.5)	12.6 (15.8)	−3.589 (3.823)	.35	0.036^a^	−5.824 (4.083)	.15	0.025^a^	−2.235 (4.515)	.62	0.047^a^
12	14.9 (24.2)	15.9 (30.8)	15.6 (20.2)	12.2 (14.9)	−3.715 (4.246)	.38	0.039^a^	−3.411 (3.332)	.31	0.031^a^	0.304 (4.315)	.94	0.050^a^
18	14.0 (25.1)	16.8 (29.1)	14.4 (26.4)	8.0 (8.7)	−8.823 (3.747)	.02	0.008^a^	−6.383 (3.647)	.08	0.017^a^	2.440 (4.830)	.61	0.044^a^
24	10.9 (16.6)	13.7 (21.0)	10.2 (12.8)	6.4 (10.3)	−7.241 (3.032)	.02	0.006^a^	−3.766 (2.382)	.11	0.019^a^	3.475 (2.964)	.24	0.028^a^
36	12.3 (20.0)	15.6 (25.9)	12.0 (15.6)	6.6 (10.7)	−9.030 (3.628)	.01	0.003^a^	−5.389 (2.720)	.048	0.014^a^	3.641 (3.805)	.34	0.033^a^

Abbreviations: CHAMP, Childhood and Adolescent Migraine Prevention; FDR, false discovery rate; NA, not applicable; PedMIDAS, Pediatric Migraine Disability Scale.

^a^ *P* value greater than FDR.

Table 3 provides PedMIDAS scores by study group and for the overall follow-up survey sample. After FDR was applied, no statistically significant results were observed.

### Current Use of Prescription Preventive Headache Medication

Reported use of prescription migraine preventive medications at each of the follow-up survey time points is shown in Table 4. Use of prescription preventive headache medication varied. The highest rate of prescription drug use was in the topiramate group at the 3-month survey, at 6 of 78 participants (8%). At several time points and in different groups, no participants reported prescription drug use, including at the 6-month survey, at which no participants reported any preventive prescription drug use. At the 3-year survey, only 1 of 153 participants (1%) reported any preventive prescription drug use (Table 4). After FDR was applied, no statistically significant results were observed.

**Table 4.** Descriptive Statistics and Statistical Analyses for Prescription Preventive Medication Use by Time Point and Group

Time point, mo	Participants, No.	Treatment group, No./total No. (%)				*P* value		
		Overall^a^	Amitriptyline	Topiramate	Placebo	Topiramate vs placebo	Amitriptyline vs placebo	Amitriptyline vs topiramate
3	203	9/203 (4)	3/80 (4)	6/78 (8)	0/45	.06	.19	.29
6	188	0/188	0/76	0/69	0/43	NA	NA	NA
12	180	3/180 (2)	2/74 (3)	1/66 (2)	0/40	.44	.29	.62
18	163	5/163 (3)	3/67 (5)	1/60 (2)	1/36 (2)	.72	.67	.37
24	163	3/163 (2)	2/70 (3)	1/57 (2)	0/36	.42	.30	.69
36	153	1/153 (1)	1/64 (2)	0/53	0/36	NA	.45	.36

Abbreviation: NA, not applicable.

^a^ Calculated as pairwise χ^2^ test with false discovery rate family-wise type-1 error correction.

## Discussion

This survey study, conducted as follow-up to the CHAMP trial,^16^ found that children and adolescents with longstanding (ie, approximately 5.7 years) and frequent migraines experienced and maintained meaningful improvements in headache status for up to 3 years after treatment. In this study, headache days (a reduction of 5 per 28 days; moving from approximately 3 per week to approximately 1.5 per week) and disability (reduced from a grade of moderate to low mild on the PedMIDAS^25,26^) improved. Use of prescription preventive medication was reported by less than 10% of participants (range, 0%-8%) throughout 3 years of follow-up after the completion of the CHAMP trial, with most participants reporting no medication use at most time points; this trend in not taking prescription preventive pharmacotherapies was generally consistent at each survey time point. No statistically significant differences were found between the CHAMP treatment groups. Given this, meaningful clinical improvement does not appear to be associated with the pharmacological action of preventive medication but rather by other mechanisms, such as the expectation of response or fluctuations of disease severity over the lifespan that may occur for some individuals with a recurrent pain condition.^30^ It remains unclear how and why youth with migraine improve and maintain these improvements over time with pharmacotherapy interventions, with the placebo effect, and with nonpharmacological interventions, such as cognitive behavioral therapy. To address these questions, future research should investigate whether neurobiological or pain processing changes, functional changes in brain activity, psychological factors, or treatment expectation changes that can result from an intervention are unique to a particular treatment.^31,32^

We could not find data from another randomized clinical trial of a prevention medication or device targeting migraine in the adult or pediatric literature that included a longitudinal follow-up survey of study participants. One comparative effectiveness trial of cognitive behavioral therapy plus amitriptyline and education plus amitriptyline for youth with chronic migraine did show maintenance of treatment gains up to 1 year.^24^ In another study examining a standardized intervention approach, outcomes at 1, 2, and 5 years were reported.^23^ In this observational investigation, a multidisciplinary treatment approach comparable with that used in the CHAMP trial^20^ demonstrated sustained clinical improvement for patients in an open-label design. This biopsychosocial approach included a thorough diagnostic interview and associated explanation of diagnosis to the patient and family, an evidence-based acute treatment plan, an adherence promotion intervention, and behaviorally focused education of healthy habits, such as increased water intake, regular meals, age-appropriate sleep schedules, and routine exercise. Although the CHAMP follow-up survey was limited by its relatively small size and inability to contact every trial participant, the rigor it used exceeds other attempts to examine outcomes over time, in that it involved participants who could return to standard clinical care and those who chose to stop seeing health care practitioners and did not continue contact with research staff from their CHAMP trial site.

In terms of study methods, the CHAMP follow-up survey is unique because it is one of the first to systematically collect data after a pharmacological migraine prevention trial for children or adults, be planned and designed before initiation of the parent clinical trial, and use technology to enhance outreach to and retention of trial participants. The retention rate of participants up to 3 years after trial completion was high, and the follow-up sample was found to be representative of all trial participants. In addition to the aforementioned strengths, the use of an internet-based assessment protocol and remote consent processes increased study feasibility and participant convenience. This application of secure data collection technology and regulatory innovation for obtaining consent/assent allowed for efficient inclusion of many sites across the United States and ongoing connection with participants in an age range that can be transitory after participation in a trial (eg, some participants may have moved from their parents’ home to college or their own homes, some families may have moved to other parts of the United States or other countries, some families stopped seeing clinicians at the trial site or in that health care system). Importantly, the resulting findings establish proof of concept such that long-term outcome assessment should be an integral process in future treatment studies in children and adults with migraine. If follow-up data are routinely obtained, comparisons across trials can occur and advances in care can be accelerated.

Clinicians should leverage potential expectation effects and use a multidisciplinary model that incorporates this expectation with acute treatment, healthy lifestyle practices, and headache education to help improve the lives of children and adolescents with migraine and their families.^20,23,32,33^ This therapeutic approach takes time, commitment, and skilled communication, and exceeds the minimal effort of writing a prescription.^34,35,36^

### Limitations

This study has some limitations. Some limitations due to this specific follow-up survey design include potential for sampling bias owing to lack of recruitment or retention of less motivated participants, relying on a self-report of the PedMIDAS measure (as opposed to using the standard structured interview approach), lack of a formal assessment of adherence to therapies (if any) during the follow-up period, and our lack of additional confirmation of medication prescriptions through external measures, such as pharmacy records. It should also be noted that we used a retrospective report of headache days vs the ongoing prospective daily diary at CHAMP baseline and throughout the CHAMP trial period; this limitation should be considered when interpreting comparisons between CHAMP study time periods. It is important to consider alternative explanations for the trends we found because this is an observational survey (eg, regression to the mean, natural disease process fluctuations); however, clinicians should also view the data in the context that participants in this survey had a mean history of headaches of approximately 6 years prior to enrollment. Additionally, these findings are from a sample of youth who chose to participate in a research study. Future study of the generalizability of these findings to individuals who seek typical clinical care (either with a headache specialist, general neurologist, or primary care practitioner) should be conducted via quality improvement and implementation science.

## Conclusions

The findings of this survey study suggest that youth with migraine who experience improvements with pill-based preventive therapy (including placebo) may maintain clinically meaningful improvements in headache days and migraine-related disability. In this study, they did so without continued use of pill-based, prescription preventive therapy. These results suggest that we should expect youth to improve, and that they continue to do well up to 3 years after pharmaceutical intervention. For clinicians, the implication of this unique trial follow-up survey study is that once an individual’s headache frequency and disability has improved, this change may be continued and sustained without future pharmacological treatment or health care utilization.

## References

[1] Abu-Arefeh I, Russell G. Prevalence of headache and migraine in schoolchildren. BMJ. 1994;309(6957):765-769. doi:10.1136/bmj.309.6957.765 7950559 PMC2541010

[2] Stewart WF, Linet MS, Celentano DD, Van Natta M, Ziegler D. Age- and sex-specific incidence rates of migraine with and without visual aura. Am J Epidemiol. 1991;134(10):1111-1120. doi:10.1093/oxfordjournals.aje.a116014 1746521

[3] Stewart WF, Lipton RB, Celentano DD, Reed ML. Prevalence of migraine headache in the United States: relation to age, income, race, and other sociodemographic factors. JAMA. 1992;267(1):64-69. doi:10.1001/jama.1992.03480010072027 1727198

[4] Bille B. A 40-year follow-up of school children with migraine. Cephalalgia. 1997;17(4):488-491. doi:10.1046/j.1468-2982.1997.1704488.x 9209767

[5] GBD 2015 Neurological Disorders Collaborator Group. Global, regional, and national burden of neurological disorders during 1990-2015: a systematic analysis for the Global Burden of Disease Study 2015. Lancet Neurol. 2017;16(11):877-897. doi:10.1016/S1474-4422(17)30299-5 28931491 PMC5641502

[6] AMF. 2017. Accessed December 8, 2020. https://americanmigrainefoundation.org/move-against-migraine/

[7] GBD 2016 Disease and Injury Incidence and Prevalence Collaborators. Global, regional, and national incidence, prevalence, and years lived with disability for 328 diseases and injuries for 195 countries, 1990-2016: a systematic analysis for the Global Burden of Disease Study 2016. Lancet. 2017;390(10100):1211-1259. doi:10.1016/S0140-6736(17)32154-2 28919117 PMC5605509

[8] Shapiro RE. What will it take to move the needle for headache disorders: an advocacy perspective. Headache. 2020;60(9):2059-2077. doi:10.1111/head.13913 32813900

[9] Parikh SK, Young WB. Migraine: stigma in society. Curr Pain Headache Rep. 2019;23(1):8. doi:10.1007/s11916-019-0743-7 30739216

[10] Steiner TJ, Stovner LJ, Vos T, Jensen R, Katsarava Z. Migraine is first cause of disability in under 50s: will health politicians now take notice? J Headache Pain. 2018;19(1):17. doi:10.1186/s10194-018-0846-2 29468450 PMC5821623

[11] Hershey AD, Oskoui M, Pringsheim T, . New guidelines: interpretation, application and the future. Headache. 2019;59(8):1133-1143. doi:10.1111/head.13629 31529478

[12] Le K, Yu D, Wang J, Ali AI, Guo Y. Is topiramate effective for migraine prevention in patients less than 18 years of age: a meta-analysis of randomized controlled trials. J Headache Pain. 2017;18(1):69. doi:10.1186/s10194-017-0776-4 28721545 PMC5515721

[13] El-Chammas K, Keyes J, Thompson N, Vijayakumar J, Becher D, Jackson JL. Pharmacologic treatment of pediatric headaches: a meta-analysis. JAMA Pediatr. 2013;167(3):250-258. doi:10.1001/jamapediatrics.2013.508 23358935 PMC4692044

[14] Powers SW, Hershey AD, Coffey CS; CHAMP Study Group. The Childhood and Adolescent Migraine Prevention (CHAMP) study: “What do we do now?”. Headache. 2017;57(2):180-183. doi:10.1111/head.13025 28128463

[15] Oskoui M, Pringsheim T, Billinghurst L, . Practice guideline update summary: Pharmacologic treatment for pediatric migraine prevention: report of the Guideline Development, Dissemination, and Implementation Subcommittee of the American Academy of Neurology and the American Headache Society. Neurology. 2019;93(11):500-509. doi:10.1212/WNL.0000000000008105 31413170 PMC6746206

[16] Powers SW, Coffey CS, Chamberlin LA, ; CHAMP Investigators. Trial of amitriptyline, topiramate, and placebo for pediatric migraine. N Engl J Med. 2017;376(2):115-124. doi:10.1056/NEJMoa1610384 27788026 PMC5226887

[17] Hershey AD, Powers SW, Bentti AL, Degrauw TJ. Effectiveness of amitriptyline in the prophylactic management of childhood headaches. Headache. 2000;40(7):539-549. doi:10.1046/j.1526-4610.2000.00085.x 10940092

[18] Winner P, Pearlman EM, Linder SL, Jordan DM, Fisher AC, Hulihan J; Topiramate Pediatric Migraine Study Investigators. Topiramate for migraine prevention in children: a randomized, double-blind, placebo-controlled trial. Headache. 2005;45(10):1304-1312. doi:10.1111/j.1526-4610.2005.00262.x 16324162

[19] Lakshmi CV, Singhi P, Malhi P, Ray M. Topiramate in the prophylaxis of pediatric migraine: a double-blind placebo-controlled trial. J Child Neurol. 2007;22(7):829-835. doi:10.1177/0883073807304201 17715274

[20] Hershey AD, Powers SW, Coffey CS, Eklund DD, Chamberlin LA, Korbee LL; CHAMP Study Group. Childhood and Adolescent Migraine Prevention (CHAMP) study: a double-blinded, placebo-controlled, comparative effectiveness study of amitriptyline, topiramate, and placebo in the prevention of childhood and adolescent migraine. Headache. 2013;53(5):799-816. doi:10.1111/head.12105 23594025 PMC3637406

[21] Powers SW, Hershey AD, Coffey CS, . The Childhood and Adolescent Migraine Prevention (CHAMP) study: a report on baseline characteristics of participants. Headache. 2016;56(5):859-870. doi:10.1111/head.12810 27039826 PMC5050048

[22] Orr SL, Kabbouche MA, O’Brien HL, Kacperski J, Powers SW, Hershey AD. Paediatric migraine: evidence-based management and future directions. Nat Rev Neurol. 2018;14(9):515-527. doi:10.1038/s41582-018-0042-7 30038237

[23] Kabbouche MA, Powers SW, Vockell AL, . Outcome of a multidisciplinary approach to pediatric migraine at 1, 2, and 5 years. Headache. 2005;45(10):1298-1303. doi:10.1111/j.1526-4610.2005.00261.x 16324161

[24] Powers SW, Kashikar-Zuck SM, Allen JR, . Cognitive behavioral therapy plus amitriptyline for chronic migraine in children and adolescents: a randomized clinical trial. JAMA. 2013;310(24):2622-2630. doi:10.1001/jama.2013.282533 24368463 PMC4865682

[25] Hershey AD, Powers SW, Vockell AL, LeCates S, Kabbouche MA, Maynard MK. PedMIDAS: development of a questionnaire to assess disability of migraines in children. Neurology. 2001;57(11):2034-2039. doi:10.1212/WNL.57.11.2034 11739822

[26] Hershey AD, Powers SW, Vockell AL, LeCates SL, Segers A, Kabbouche MA. Development of a patient-based grading scale for PedMIDAS. Cephalalgia. 2004;24(10):844-849. doi:10.1111/j.1468-2982.2004.00757.x 15377315

[27] Snijders TA, Bosker RJ. Multilevel Analysis: An Introduction to Basic and Advanced Multilevel Modeling. 2nd ed. Sage; 2012.

[28] Benjamini Y, Hochberg Y. Controlling the false discovery rate: a practical and powerful approach to multiple testing. J R Stat Soc B. 1995;57(1):289-300. doi:10.1111/j.2517-6161.1995.tb02031.x

[29] Headache Classification Committee of the International Headache Society (IHS). The International Classification of Headache Disorders, 3rd edition. Cephalalgia. 2018;38(1):1-211. doi:10.1177/0333102417738202 29368949

[30] Cormier S, Lavigne GL, Choinière M, Rainville P. Expectations predict chronic pain treatment outcomes. Pain. 2016;157(2):329-338. doi:10.1097/j.pain.0000000000000379 26447703

[31] Institute of Medicine (US) Committee on Advancing Pain Research, Care, and Education. Relieving Pain in America: A Blueprint for Transforming Prevention, Care, Education, and Research. National Academies Press; 2011. 22553896

[32] Nahman-Averbuch H, Schneider VJ II, Chamberlin LA, . Alterations in brain function after cognitive behavioral therapy for migraine in children and adolescents. Headache. 2020;60(6):1165-1182. doi:10.1111/head.13814 32323877

[33] Faria V, Linnman C, Lebel A, Borsook D. Harnessing the placebo effect in pediatric migraine clinic. J Pediatr. 2014;165(4):659-665. doi:10.1016/j.jpeds.2014.06.040 25063720 PMC4358740

[34] Ernst MM, O’Brien HL, Powers SW. Cognitive-behavioral therapy: how medical providers can increase patient and family openness and access to evidence-based multimodal therapy for pediatric migraine. Headache. 2015;55(10):1382-1396. doi:10.1111/head.12605 26198185 PMC4715506

[35] Lipton RB, Hahn SR, Cady RK, . In-office discussions of migraine: results from the American Migraine Communication Study. J Gen Intern Med. 2008;23(8):1145-1151. doi:10.1007/s11606-008-0591-3 18459012 PMC2517978

[36] Buse DC, Lipton RB. Facilitating communication with patients for improved migraine outcomes. Curr Pain Headache Rep. 2008;12(3):230-236. doi:10.1007/s11916-008-0040-3 18796275

